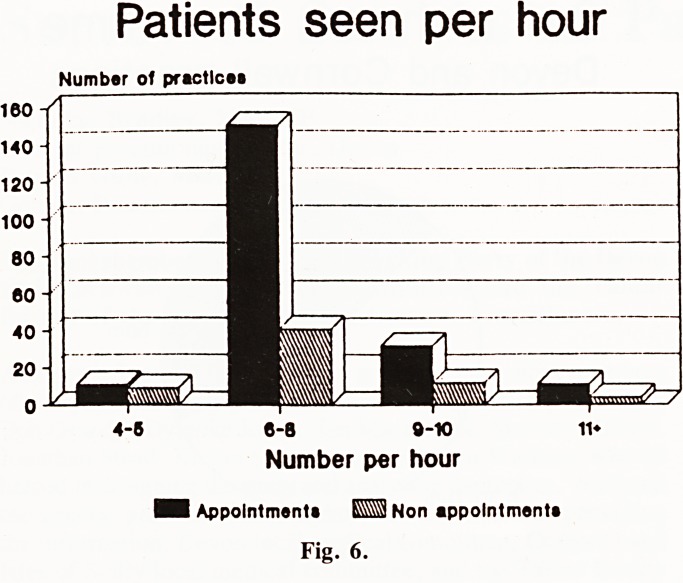# Survey of Services to Patients in General Practice

**Published:** 1992-03

**Authors:** Nicholas Bradley, Rupert Gude

**Affiliations:** General Practitioner, Exeter, Devon; General Practitioner, Tavistock, Devon


### Survey of Services to Patients in General Practice

**Nicholas Bradley, MRCGP**
General Practitioner, Exeter, Devon
**Rupert Gude, MRCGP**
General Practitioner, Tavistock, Devon

**Both are members of the Joint Working Party of the Devon and Cornwall local medical committees and the Tamar faculty of the Royal College of General Practitioners.**

**Acknowledgements:** Our thanks go to the nine other members of the joint working party: Michael Ellis, Christopher Fagg, Bob Grundy, Dymoke Jowitt, Ian MacKenzie, Stephen Millard, Jonathan Stead, Kieran Sweeney and Stephen Watkins, who all helped in designing the study and analysing the replies. We thank the general practitioners of Devon and Cornwall for providing the information; Devon local medical committee, Cornwall and Isles of Scilly local medical committee, and the Tamar faculty of the Royal College of General Practitioners for funding the study; Sarah Lane, Valerie Harris and Imelda Liversage for their secretarial help; Leo Clarke for the computer graphics; and Ottery Press, Tavistock for printing.

### SUMMARY

Family doctors vary in the range of services they provide for their patients. Of 267 practices in Devon and Cornwall, 245 responded to a questionnaire sent in September 1989 asking for information about services to patients. Most doctors consulted at six to eight patients per hour, whether or not an appointment system operated. About two-thirds of patients had access to a female GP. Nine out of ten practices employed a practice nurse. Almost all offered a surgery on Saturday mornings. Most surgeries took the phone over at 8.30 a.m. in the week and started consulting at 9 a.m. Almost half were consulting until 6.30 p.m. or later on at least one day per week. 80% of practices were offering a non-urgent appointment on the same or next day. All practices offered childhood immunisation. 80% offered some form of personal list system. 80% offered minor operations, one-third manipulations, 10% homeopathy, 6% hypnosis and 5% acupuncture.

### INTRODUCTION

There is a wide variation in styles of general practice and the amount of money that family doctors invest in their practices.[1] Some doctors consult fast, others slowly, some provide easier access than others, some practices work with practice nurses, attached health visitors and physiotherapists, and offer skills like manipulation or minor surgery. It is difficult for a family doctor to know how his style of practice compares with that of other doctors. The joint working party of the Devon and Cornwall local medical committees and the Tamar faculty of Royal College of Practitioners has over the last five years looked at aspects of how general practitioners in Devon and Cornwall work and has shared its pooled results with all the practices in the two counties. It has published work for strategies on recording smoking and blood pressure,[2] on recording preventive care activities,[3] and on practice equipment.[4] The aim of this study was to document the range of services available to patients in Devon and Cornwall in the Autumn of 1989 and to feed back this information to all practices as a focus for discussion and development.

### METHODS

All the practices in Devon and Cornwall received an anonymous postal questionnaire in September 1989. Non-responders, identified by a code number on the questionnaire, received reminders on up to two occasions at monthly intervals. Only the study secretary held the code list which was destroyed after analysis. The rubric suggested that the senior receptionist in each practice would be the most appropriate person to answer the questions. We asked about the list size and the types of doctors and other clinical staff working in the practice; whether lists were "shared" or "personal"; about the timing and arrangements of surgeries and on call out-of-hours cover; about the kinds of extra services and skills that were available to patients; and about how the practices communicated with patients, for example by way of leaflets, telephones, groups, reports or libraries. In September 1990, every general practitioner in the two counties received a printed report of the results of the survey.

### RESULTS

**Response.** 245 practices (91.8%) returned unspoiled anonymous questionnaires. Some questions depended on the respondent answering for an average day. Answers reflected what they thought the practice offered.

**Distribution of practices and patients by list size (Fig. 1)** We banded practices by list size (in thousands) into small (1,000 to 2,999), medium (3,000 to 5,999), large (6,000 to 10,999) and grand (11,000 plus) practices for the purpose of analysing results. About one in ten practices in Devon and Cornwall are single-handed. Small practices accounted for about one quarter of the practices in the area, but were looking after less than 10% of the population. The handful of grand practices catered for about one quarter of the population.

**Fig. 1**

**Categories of doctors working in practices. (Table 1).** One-third of practices had trainees, yet in small practices there were only three trainees in 63 practices. Half the larger practices had trainees. 61% of practices had female doctors and these practices served 69% of the population. A quarter of the large and grand practices did not have a female doctor.

**Staff working in practice premises (Table 2).** There is considerable variation in the availability of different staff from practice to practice. This is because many attached staff work with the practice but do not share the practice premises. Practice nurses worked in 92% of practices. A broad range of professionals were working in practices and a number of hospital based specialists offered facilities from practice premises.

**Morning start times. (Fig. 2).** About a quarter of practices took the telephone over from the night service at 8 a.m. or 8.15 a.m. and one-third started surgeries at 8.30 a.m. or before. Most surgeries took the telephone over at 8.30 a.m. and started consulting at 9 a.m. 80% of practices opened the door to patients when the phone was taken over. 49 surgeries (20%) delayed opening the door for 30 minutes. One quarter of practices said they started their first consultation at the same time as the doors were opened for patients. This arrangement must mean that patients sometimes have to wait for admission to practice premises.

Fig. 2

**Consulting Times (Fig. 3).** There was a big variation from practice to practice. Some had surgeries running throughout the day. Some had only morning and evening surgeries. A third of practices started at 8.30 a.m. with nearly all consulting between 9 a.m and 11 a.m. 60% of practices had surgeries at 11 a.m. to 12 noon, and a quarter at 12 a.m. to 1 p.m. 16% of practices closed the premises completely for half a weekday per week.

Fig. 3.

**Out of hours. (Fig. 4).** Most practices put the phone over at 6.30 or 7 p.m., although a third did this at 6 p.m. or earlier. Half used an answerphone for at least one of their partners. This was more common in the smaller practices. 18% of practices used a deputising service. This varied from an occasional night to 21 days in a month.

Fig. 4.

**Saturday surgery. (Fig. 5).** 95% of practices offered a Saturday morning surgery. One in ten small practices considered that Saturday surgeries were for routine appointments as well as urgent problems. Three quarters of practices felt that they were for urgent problems only. One fifth of all practices and a third of the small/medium practices saw five patients or less on a Saturday. Most (61%) saw between 15 and 16 patients. We compared the numbers seen to list size. Most practices (62%) saw between 0.6 and 2.0 patients per thousand registered. Small practices saw a larger proportion of the patients on their list on a Saturday morning.

Fig. 5.

**Type of surgeries. (Table 3).** Non-appointment systems are more common in smaller practices, but half of them had appointment systems. Three of the large practices had a non-appointment system.

**Time to obtain an appointment.** All practices said they would see an urgent case the same day. 80% would offer an appointment for non-urgent cases on the same or next day. This was more common in smaller practices. In 6% there was a delay of four days or more for a non-urgent appointment.

**Length of consultations. (Fig. 6).** Two-thirds of practices booked six to eight patients per hour. 10% booked 10 per hour and 5% 12 per hour. Five practices gave 15 minute appointments. With non-appointment systems, 60% of practices still saw 6-8 patients per hour, 13% seeing 4-5 patients per hour. Non-appointment surgery sessions resulted in an almost identical pattern of consulting time to those run by appointment.

Fig. 6.

**Type of list.** See **Table 4.**

**New patients.** 35% of practices always offered an introductory consultation with the doctor. 10% used a nurse to fulfill this function.

**Child health.** All practices offered child immunisations. In 55% they were done by the practice nurse; in 24% by the partner only, and more commonly in smaller practices; in a quarter they were done by either the doctor or the nurse. 53% held a regular well baby clinic. 48% ran their own child surveillance.

**Skills. (Table 5).** The skills most commonly offered appear in table 5. Other skills listed in three or less practices were: relaxation, counselling, psycho-sexual counselling, vasectomy, cryotherapy and glaucoma checks.

**Communication with patients.**

- Practice leaflets were available in 69% of practices: 52% of smaller practices, 92% of the largest practices.
- 23% of practices ran patient participation groups.
- 28% had patient libraries.
- 8% of practices had an annual report for their patients, 29% a report for their partners.
- 87% of practices had access for wheel chairs.
- One quarter of practices stocked batteries and leads for hearing aids.
- 65% of practices had a defined time for telephone advice.

### DISCUSSION

We surveyed 245 out of 267 practices on the Family Practitioner Committee (now the Family Health Services Authority) lists in Devon and Cornwall on the services they were offering to their patients, in autumn 1989. The results relate to services involving 954 individual doctors catering for some 1.5 million people as patients. They reflect the "pre-contract" position.

Patients do express concerns about difficulty in getting to see their doctors.[5] All practices in our study offered appointments for urgent problems the same day, and it was encouraging to see that 80% of them would see a patient with a non-urgent problem on the same or next day. One-third of practices started surgeries at 8.30 a.m. and almost half were consulting to 6.30 p.m. or later at some time in the week, providing a spread of times for patients to consult. There was great variation between practices: some offered very limited hours of availability and some partnerships closed their premises for half a weekday per week.

There is also concern that appointment systems restrict access to doctors[6] and yet there is considerable support for personal lists to provide continuity of care. Priestman argues that some compromise is inevitable:[7] 55% of practices in this study had a personal list system with the facility for patients to consult another doctor, if necessary. An appointment system can be a barrier, preventing patients getting at their doctor:[8] a third of practices in Devon and Cornwall tackle this by running a mixed system of appointments and open access surgeries.

How much time do patients get with the doctor when they do get to see him or her? 60% of practices in our study said that they book 6-8 patients per hour. Anderson and Buxton, in a survey of 125 doctors in Greenwich,[9] asked how many patients a general practitioner saw in an hour. They found this distribution: 5 or 6 — 10%; 7 or 8 — 29%; 9 or 10 — 29%; and 11 or 12 — 22%. Equivalent booking figures in our study were: 4, 5 or 6 — 38%; 7 or 8 — 36%; 9 or 10 — 19% and 11 or over — 5%. The distribution was the same for practices with and without appointment systems.

Wilson asked general practitioners what rate of consulting was compatible with practising to their highest standard.[10] The median response was 6 patients per hour. A third of Devon and Cornwall practices seem to be achieving this standard.

In a survey in North-West England in 1985, Allen[5] found a strong desire for access to a general practitioner by telephone. Two-thirds of practices in Devon and Cornwall provide a defined time for telephone advice.

Most doctors felt that Saturday surgeries were for urgent problems only. 10% of smaller practices but only one larger practice offered routine appointments on a Saturday. Two larger practices did not have a Saturday surgery at all. 24 practices saw 3 patients or less on the particular Saturday that was reported. We have no information as to whether this was usual or abnormally low. In general, smaller practices saw a greater proportion of the patients on their list on a Saturday than larger practices.

There are some areas in which the new contract will be bound to have forced changes upon a number of practices, judging by our results. Only 35% of practices offered an introductory consultation with a newly registered patient: this is now required by the contract. All practices have had to compile an annual report for their Family Health Services Authority (FHSA) by the end of June 1991: less than one third had done this in our study. 21% of practices will have had to produce "de novo" a leaflet for patients since the new contract.

In general there seemed to be a high quality of service to patients, reflected in the early start times, long consultations, convenient consulting hours, thoughtful appointment systems, widespread access to female doctors, low use of deputizing services, all practices doing childhood immunisations, and a rich variety of additional services such as practice nurses, minor surgery and manipulation.

With the new contract has come a greater expenditure by certain FHSA's on general medical services than was ever anticipated. Health service managers must appreciate that high cost general medical services may reflect high pre-contract levels of activity and patient care.

Our study provides evidence, too, of general practitioners' willingness to take part in practical medical audit when they see that the data will be used helpfully and fed back direct to them. 91% of practices performed this simple audit task to delineate a set of normative standards for future improvements in Devon and Cornwall.

Correspondence to: Dr. N. C. A. Bradley, The Surgery, 12 Lovelace Gardens, Alphington, Exeter EX2 8XQ.

### REFERENCES

1. BOSANQUET N, LEESE B. Family doctors: their choice of practice strategy. *Br.Med.J.* 1986: **293:** 667-670.
2. Joint working party Devon and Cornwall LMC, and Tamar faculty RCGP. Recording blood pressure and smoking habits in Devon and Cornwall. Exeter: Heriz Studios; 1986.
3. GRUNDY R, DWYER D. Preventive care card for general practice. *JR. Coll. Gen. Pract.* 1989; **39:** 15-16.

4. BRADLEY N, WATKINS S. Survey of equipment in general practice. *Br.Med.J.* 1989; **299:** 435-436.
5. ALLEN D, LEAVEY R, MARKS B. Survey of patients' satisfaction with access to general practitioners. *JR. Coll. Gen. Pract.* 1988; **38:** 163-165.
6. ARBER S, SAWYER L. Do appointment systems work? *Br. Med.J.* 1982; **284:** 478-480.
7. PRIESTMAN S. Personal versus shared lists: a continuing debate. *JR. Coll. Gen. Pract.* 1987; **37:** 147-148.
8 FREEMAN G. Receptionists, appointment systems and continuity of care. *JR. Coll. Gen. Pract.* 1989; **39:** 145-147.
9. ANDERSON R, BUXTON A. Consultation length: general practitioners' attitudes and practices. *Br. Med.J.* 1985; **290:** 1903.
10. WILSON A. Consultation length: general practitioners' attitudes and practices. *Br. Med.J.* 1985; **290:** 1322-1324.

**Table 1**

| | Number of doctors in general practice in Devon and Cornwall |
|---|---|
| Full time partners | 723 |
| Part time partners | 102 |
| Trainees | 82 |
| Other doctors | 47 |
| TOTAL | 954 |

**Table 2**

**Types of non-clerical staff working on practice premises**

| | Number of practices (n=245) | | Number of practices (n=245) |
|---|---|---|---|
| Practice nurse | 226 | Community nurse | 122 |
| Health visitor | 91 | Midwife | 201 |
| Behaviour therapist | 28 | Counsellor | 31 |
| Physiotherapist | 33 | Chiropodist | 66 |
| Speech therapist | 43 | Social worker | 16 |
| Child psychiatrist | 4 | Psychiatrist | 15 |
| Chest physician | 1 | Orthoptist | 4 |
| Audiologist | 2 | Dietician | 4 |

**Table 3**

**Type of surgeries**

| | Number of practices (n=245) |
|---|---|
| Appointments only | 154 |
| Mixed | 73 |
| Non appointment | 18 |

**Table 4**

**Type of list**

| | Number of practices (n=211) |
|---|---|
| Personal list with: | |
| (a) patient having to see own doctor | 51 |
| (b) facility to consult others | 117 |
| No personal list | 43 |

(Not all respondents answered this question)

**Table 5**

**Skills**

The following skills were offered:

| | Number of practices (n=245) |
|---|---|
| Minor operations | 196 |
| Joint injections | 201 |
| Manipulations | 75 |
| Homeopathy | 24 |
| Acupuncture | 12 |
| Hypnosis | 15 |

## Figures and Tables

**Fig. 1 f1:**
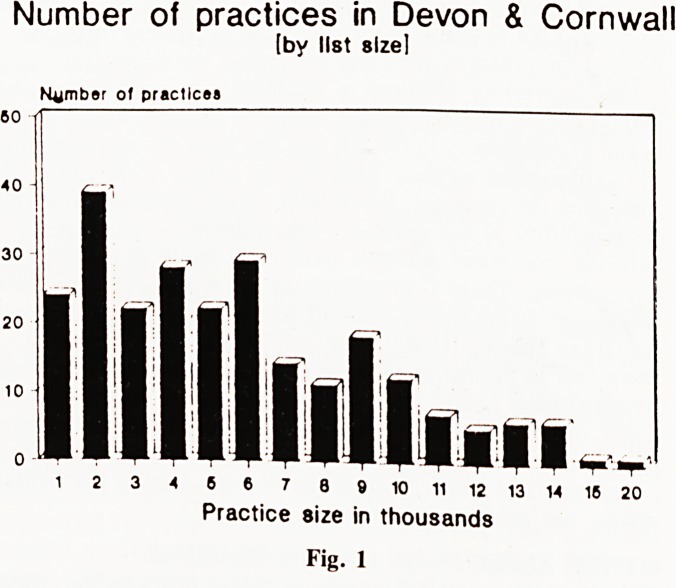


**Fig. 2 f2:**
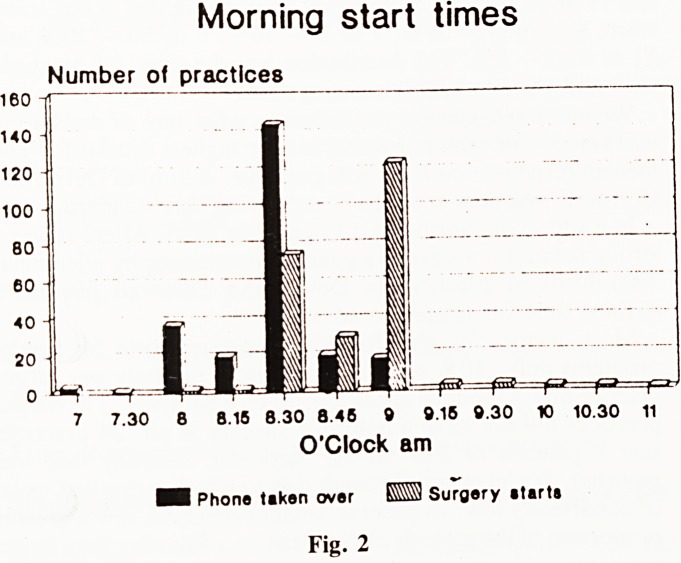


**Fig. 3 f3:**
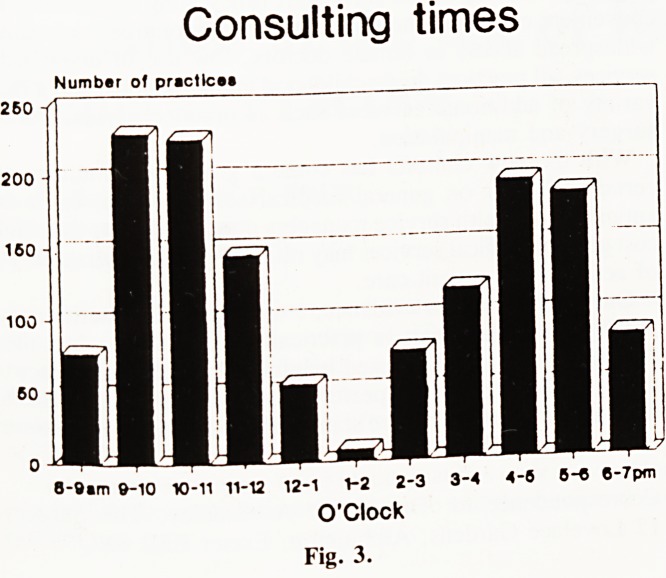


**Fig. 4. f4:**
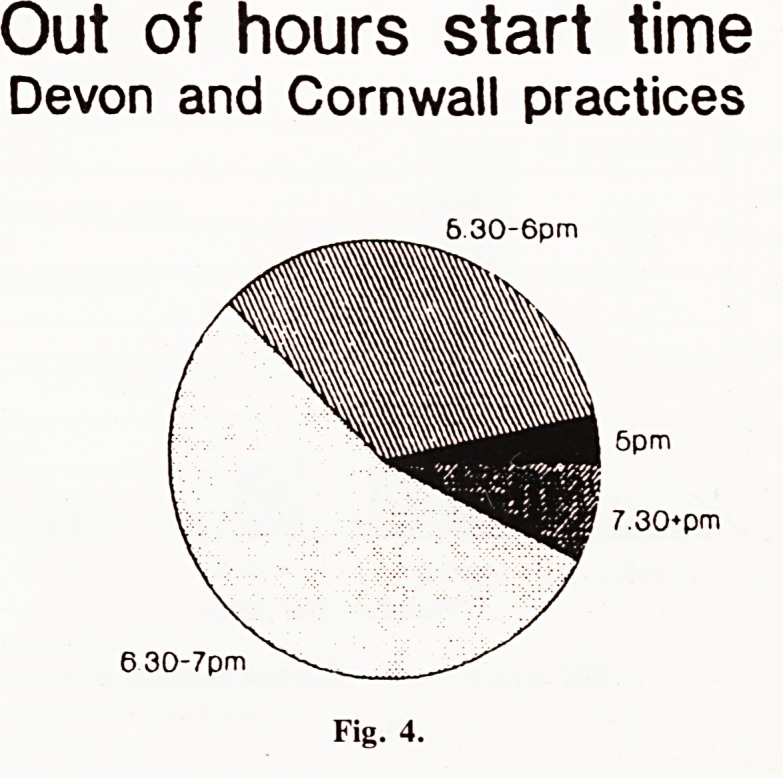


**Fig. 5. f5:**
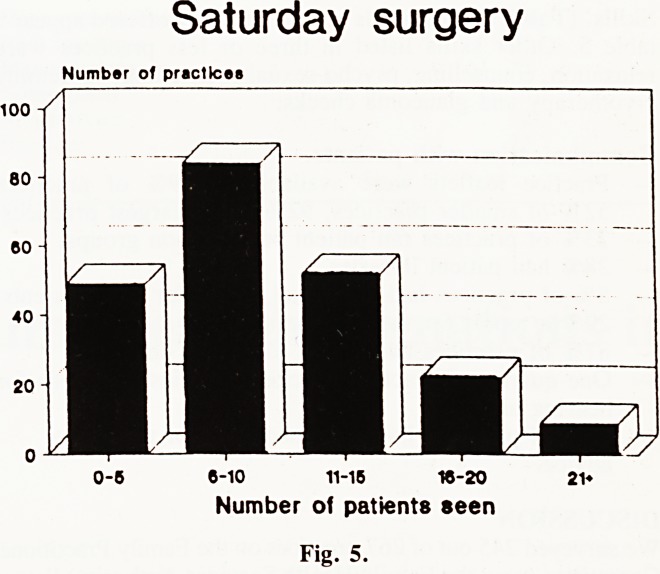


**Fig. 6. f6:**